# The Mammalian Peptide Adrenomedullin Acts as a Growth Factor in Tobacco Plants

**DOI:** 10.3389/fphys.2017.00219

**Published:** 2017-04-12

**Authors:** Rafael Peláez, María Niculcea, Alfredo Martínez

**Affiliations:** ^1^Biomass Booster LtdLogroño, Spain; ^2^Oncology Area, Center for Biomedical Research of La RiojaLogroño, Spain

**Keywords:** adrenomedullin, *Nicotiana tabacum*, growth factor, plant survival, biomass increase, cell cycle

## Abstract

Growth factors are extracellular signals that regulate cell proliferation and total body mass. Some animal growth factors can work on plant tissues and vice versa. Here we show that the mammalian growth factor adrenomedullin (AM) induces growth in tobacco plants. Addition of synthetic AM resulted in a dose-dependent growth of tobacco calluses. Furthermore, AM transgenic plants showed enhanced survival and significant increases in stem diameter, plant height, leaf length, weight of all organs, and a reduction in the time to flowering when compared to plants transformed with the control vector. These differences were maintained when organs were dried, resulting in a mean total biomass increase of 21.3%. The levels of soluble sugars and proteins in the leaves were unchanged between genotypes. AM transgenic plants had a significantly higher expression of cyclin D3 and the transcription factor E2FB than controls, suggesting that cell cycle regulation may be part of the intracellular signaling of AM in plants. In summary, mammalian AM increases vascular plants' survival and biomass with no apparent detriment of plant's morphological and/or biochemical properties, thus this strategy could be useful for crop productivity improvement.

## Introduction

Growth factors are signaling molecules produced by living organisms to stimulate their cellular growth rate, healing, cellular differentiation, and final size. Chemically, growth factors are very heterogeneous and can be constituted by peptides, such as growth hormone or insulin-like growth factors in mammals (Pfaffle, 2015) or root meristem growth factor in plants (Shinohara et al., 2016); lipids such as corticosteroids (Loke et al., 2015) or strigolactones (Pandey et al., 2016); secondary metabolites such as auxins or gibberellins (Gantait et al., 2015; Li et al., 2016); or even gaseous mediators such as nitric oxide or ethylene (Napoli et al., 2013; Street et al., 2015). It is commonly understood that plants and animals are two very distinctive systematic groups and they are supposed to rely on completely different signaling molecules, but there is evidence that some molecules may have activity in both kingdoms. For instance, addition of human epidermal growth factor to sorghum seedlings (Dyer, 1980) or to corn primary roots and mesocotyls (Kato et al., 1995) resulted in a significant growth increase for these structures. In addition, the reverse situation has been reported as well, with plant peptides being able to bind and activate growth factor receptors in human cells (Treggiari et al., 2015; Molesini et al., 2017). A common signaling molecule is nitric oxide, which reduces growth in plants (Arora and Bhatla, 2017) and has a miscellaneous effect on mammalian cells, depending on nitric oxide concentration and cell type (Napoli et al., 2013).

Adrenomedullin (AM) is a 52 amino acid peptide with an internal disulphide bond and a terminal amide group, whose tridimensional structure is characterized by a central alpha-helix (Lopez and Martinez, 2002; Perez-Castells et al., 2012). AM is produced from a 185 amino acid precursor termed preproadrenomedullin, which undergoes post-translational processing by endo- and exo-peptidases, generating a series of chemical intermediaries. The last intermediate before fully mature AM is Gly-extended AM (or Gly-AM), which usually lacks physiological activity (Martinez et al., 2006). AM homologs have been found in all vertebrates, from humans to fish (Martinez et al., 2006), and immunoreactive epitopes were detected in invertebrates such as echinoderms (Martinez et al., 1996). No apparent homology is found between AM and any of the described plant peptide hormones. Among other physiological roles, AM has been shown to act as a potent growth factor for normal (Isumi et al., 1998) and tumor cells (Miller et al., 1996). In addition, the abrogation of the gene coding for this peptide results in 100% embryo lethality (Caron and Smithies, 2001), underscoring the relevance of this molecule for normal development in mammals.

Given the broad distribution of AM among animals and its demonstrated potency as a growth factor, we decided to investigate whether this peptide may have some influence on the *in vitro* and *in vivo* growth of vascular plants, choosing tobacco as a model organism.

## Materials and methods

### *In vitro* culture and treatment of tobacco calluses

Seeds of *Nicotiana tabacum brasiliensis*, cv Paraguay, were obtained from University of Navarra germplasm collection and cultured under standard conditions. Calluses were prepared from explants of young tobacco leaves placed in callus-producing solid medium [Murashige and Skoog (MS) complete medium (Murashige and Skoog, 1962) plus 100 mg/L 2-4D and 100 mg/L kinetin] and incubated in the dark at 24°C. When the calluses were large enough, they were divided in small fragments that were weighed and transferred to fresh callus-producing solid medium containing different amounts of AM or Gly-AM synthetic peptides (Phoenix Pharmaceuticals, Burlingame, CA), as indicated. Synthetic peptides had a purity >95%. Cultures were grown in the dark at 24°C for 30 days. At this time, individual calluses were weighed again. Growth rate was calculated as the weight at the end of the procedure divided by the weight of the same fragment at the beginning. All values were normalized to the untreated control that was given the value 100. Calluses were dried in an oven (Selecta, Barcelona, Spain) at 80°C and weighed again.

### Production of a molecular vector for transgenesis

Mammalian AM was cloned from a cDNA library obtained from human lung carcinoma cell line A549, which has been previously shown to express high levels of AM (Miller et al., 1996). PCR was performed with primers AM sense: ATG AAG CTG GTT TCC GTC GCC CTG and AM antisense: CTA AAG AAA GTG GGG AGC ACT TCC ACT CG, thus cloning a fragment containing mature AM, surrounded by restriction sites XbaI and SacI (underlined). PCR products were subcloned into pCRII vector (ThermoFisher, Madrid, Spain) and the identity of the cloned product was confirmed through direct sequencing. This plasmid was named pCRII-AM. The *A. tumefaciens* Ti-based vector pBI-121 was purchased from Clontech (Palo Alto, CA). Both pBI-121 and pCRII-AM were excised with XbaI and SacI and the AM fragment was inserted in the pBI vector in place of the GUS gene. In this vector, gene expression is driven by the 35S promoter. This new vector was named pBI-AM and its sequence fidelity was also confirmed through direct sequencing.

### Generation of transgenic tobacco plants

*Agrobacterium tumefaciens* bacteria were transformed with plasmids pBI-121 and pBI-AM by electroporation and grown in YEP medium (10 g/L yeast extract, 10 g/L peptone, 5 g/L NaCl, pH 7.0) without antibiotics at 30°C. Three hours later, bacteria were transferred to YEP medium containing 100 μg/ml streptomycin and 50 μg/ml kanamycin and incubated overnight at 30°C. Optical density of the bacterial culture was adjusted to 0.8 and small tobacco leaf fragments were immersed in this medium for 30 min and then transferred, still with the bacteria, to solid co-cultivation MS medium (with 0.6 g/L MES and 1 mg/ml BAP) without antibiotics, following a protocol slightly modified from Horsch et al. (1986). After 72 h, leaf fragments were transferred into shoot MS regeneration medium with 0.6 g/L MES, B5 vitamins, 100 mg/ml carbenicillin, 100 mg/ml cefotaxime, and 50 mg/ml kanamycin and cultured bi-weekly to the same medium. *In vitro* plant culture was performed inside an environmental chamber (FitoClima 1200 Bio, Aralab, Rio de Mouro, Portugal) in dim light (60–80 mE/m.s.) and 18/6 photoperiods at 24°C. When shoots and leaves reached a convenient size, they were transferred to root development medium (MS/B5/MES medium and antibiotics) and when the radicular system was mature enough, the plantlets were placed in small pots containing regular soil. At this point young plants of both genotypes were moved into the greenhouse and regular horticultural care was applied. Periodically, several morphological parameters such as plant height, stem diameter, number of leaves, leaf length, and internodal length were measured in all plants by researchers blinded to the actual genotypes. After the first floral buds opened but before complete flower maturation, all plants were uprooted and weighed, measuring total weight and the weight of the roots, stems, leaves, and flowers. After drying the plants, the dry weight for all organs was also recorded.

### Genotyping of transgenic plants

DNA was obtained from fresh leaf punches with the Phire Plant Direct PCR kit (Thermo Scientific). PCR was performed with primers AM sense: CGC CAG AGC ATG AAC AAC T; AM antisense: CGA CGT TGT CCT TGT CCT TA; GUS sense: TGC TGT CGG CTT TAA CCT CT; and GUS antisense: GGC ACA GCA CAT CAA AGA GA. AM primers render a PCR amplicon of 121 bp while GUS primers produce a 332 bp band. PCR was run in a GeneAmp PCR System 9700 (Applied Biosystems, Foster City, CA) for 30 cycles with an annealing temperature of 60°C.

### Gene expression quantification

Leaf tissue samples were homogenized with TRIzol (Invitrogen, Madrid, Spain) and RNA was isolated with RNeasy Mini kit (Qiagen, Germantown, MD). Three micrograms of total RNA were treated with 0.5 μl DNAseI (Invitrogen) and reverse-transcribed into first-strand cDNA using random primers and the SuperScript III kit (Invitrogen). Reverse transcriptase was omitted in control reactions, where the absence of PCR-amplified DNA confirmed lack of contamination from genomic DNA. Resulting cDNA was mixed with SYBR Green PCR Master Mix (Invitrogen) for quantitative real time polymerase chain reaction (qRT-PCR) using 0.3 μM forward and reverse oligonucleotide primers (Table 1). Quantitative measures were performed using a 7300 Real Time PCR System (Applied Biosystems, Carlsbad, CA). Cycling conditions were an initial denaturation at 95°C for 10 min, followed by 40 cycles of 95°C for 15 s and 60°C for 1 min. At the end, a dissociation curve was implemented from 60 to 95°C to validate amplicon specificity. Gene expression was calculated using absolute quantification by interpolation into a standard curve. All values were divided by the expression of the house keeping gene Tubulin α1.

**Table 1:** **Primers used for qRT-PCR**.

**Target**	**Sequence**	**Access number**
AM-F	TTCGAAAGAAGTGGAATAAGTGGG	NM_001124.2
AM-R	CCGCAGTTCCCTCTTCCC	
CYCD3-F	GATTTTCAAGTGGAGGATGCTA	AJ011893
CYCD3-R	TCAACAGAATTACAAGGCTCAAC	
GTPase-F	TGCTGCTGTTACATTCTGTTGTAGAT	EB683389
GTPase-R	GGCAACGGCCAAAAGAAA	
E2FB-F	TGCTGATACATTAGAGGTTCAGA	HE653923.1
E2FB-R	TTCTCCTGGTTTTGAGACG	
AuxResp-F	TCACCTGGGAGAGTTTATGGACTAC	DW001943
AuxResp-R	TCTGTAACATGCCCAAACTAAACG	
CDKB1-F	GGCTTTCACTGTCCCAATAA	AF289465
CDKB1-R	GAGGCCAAGTTCTGAGGTTC	
TTG2-F	GGAGTTGGAAAGGCATCAGG	FJ795022.1
TTG2-R	AATCCCATTAGGCCCAGCAA	
TUBα1-F	CAAGACTAAGCGTACCATCCA	AJ421411
TUBα1-R	TTGAATCCAGTAGGGCACCAG	

*Annealing temperature for all primers was 60°C*.

### Protein extraction and western blotting

Small leaf fragments were homogenized in RIPA buffer (Thermo Scientific) containing protease (EDTA-free complete, Roche, Basilea, Switzerland) and phosphatase (PhosStop, Roche) inhibitors, 2 mM dithiothreitol, and 8 M urea. Homogenates were sonicated three times (Soniprep 150, MSE, London, UK), centrifuged for 30 min at 15,000 × g and the supernatants collected. Protein concentration was determined with the BCA kit (Pierce, Rockford, IL), with bovine serum albumin as standard, using a spectrophotometer (POLARstar Omega, BMG Labtech, Ortenberg, Germany). Then, 100 μg of each sample were mixed with 4x sample buffer (Invitrogen) and heated for 10 min at 70°C. Samples were run on 4–12% SDS—polyacrylamide gels. Seeblue plus 2 Prestained Standards (Invitrogen) were used as molecular weight markers. For Western blot analysis, proteins were transferred onto 0.2-μm polyvinylidene difluoride (PVDF) membranes (iBlot system, Invitrogen). For protein identification, membranes were incubated overnight at 4°C with a rabbit polyclonal anti-AM antibody made in house and previously characterized (Martinez et al., 1995) at a 1:2,000 dilution. To visualize immunoreactivity, membranes were incubated with peroxidase-labeled goat anti-rabbit IgGs (Jackson Immunoresearch, West Grove, PA), developed with a chemoluminiscence kit (GE Biosciences, Miami, FL), and exposed to X-ray films (GE Biosciences).

### Histological processing

Fragments of roots, stems, and leaves were immersed in FEA fixative (5% formalin, 45% ethanol, 5% acetic acid) for 24 h, dehydrated, and embedded in paraffin. Sections (3 μm-thick) were stained with hematoxylin-eosin. Photographs were taken with an Eclipse 50i microscope (Nikon, Barcelona, Spain) equipped with a DXM 1200C digital camera (Nikon).

### Biochemical composition of transgenic plants

To investigate whether the weight differences were reached in detriment of the biochemical characteristics of the transgenic plants, we measured the concentration of starch, soluble sugars, and proteins in mature leaves of both genotypes. Soluble sugars were estimated following established methods (Yemm and Willis, 1954). Briefly, frozen leaves were reduced to powder and 250 μl of this extract were mixed with 3.0 ml anthrone reagent, stirred, and incubated at 80°C for 10 min. After cooling, absorbance was measured at 620 nm. Concentration of soluble proteins was evaluated with a BCA protein assay kit (Thermo Scientific, Rockford, IL). Starch concentration was measured as reported (Jarvis and Walker, 1993). Briefly, leaves were dissociated in 5 ml 95% ethanol and centrifuged at 28,710 × g for 10 min at 4°C. Two ml 1 M KOH were added to the supernatant and the solution was incubated in the dark for 24 h to solubilize the starch. Then, the solution was neutralized with 2 ml 1 M HCl and 0.5 ml iodine reagent were added and allowed to react for 15 min in the dark. The resulting solution was measured at 565 nm.

### Statistical analysis

Statistical analysis was performed with SPSS v18 software package. Data sets were tested for normalcy (Kolgomorov-Smirnov) and homoscedasticity (Levene). Normally distributed data were compared using Student's *t*-test or ANOVA followed by *post-hoc* tests (Dunnet). When data did not follow a normal distribution they were compared with Mann-Whitney's *U* non-parametric test. Survival rates were analyzed using a χ^2^-test. A *p*-value lower than 0.05 was considered statistically significant.

### Search for AM orthologs in tobacco's genome

The genome of *N. tabacum* is available (Sierro et al., 2014) and a search of tobacco's predicted proteome was performed looking for peptides with some sequence homology to any of the available vertebrate AM sequences (Martinez et al., 2006) using BLAST approaches, Prosite, and the EggNOG v3.0 database, using threshold default values (Karlin and Altschul, 1990), as described (Kuzniar et al., 2008). We also performed a search for the peptide sequences Cys-X-X-X-X-Cys and Tyr-Gly-Arg-Arg-Arg-Arg-Arg, which define the motifs essential for maintaining AM's function (Lopez and Martinez, 2002).

## Results

### AM induces tobacco callus growth *In vitro*

**Figure 1:**
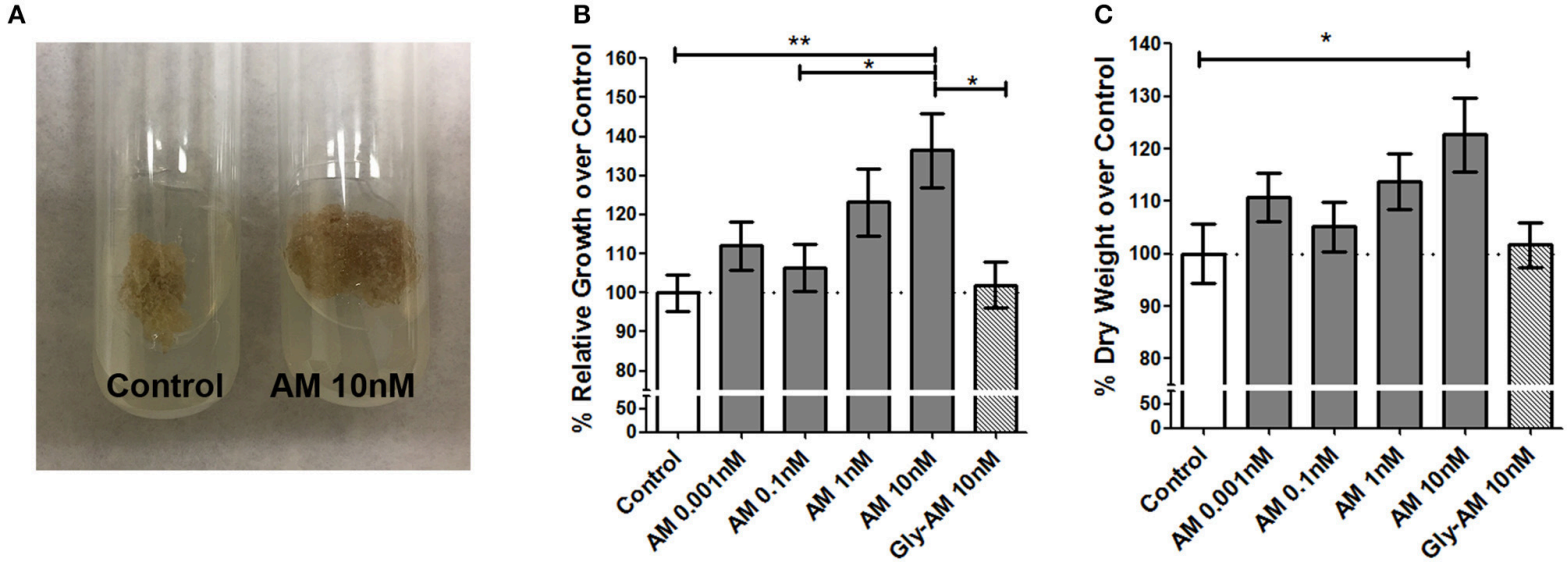
**AM induces growth in tobacco calluses**. Small tobacco callus fragments were weighed and cultured in solid growth medium containing different concentrations of synthetic AM or Gly-AM for 30 days in the dark **(A)**. At the end of this period they were weighed again. The relative growth of the calluses increased at higher doses **(B)**. After the calluses were dried, the differences in weight remained **(C)**. Gly-AM had no significant effect even at the higher concentration tested. Bars represent the mean ± *SD* of all specimens (*n* = 25 per treatment). Asterisks indicate statistically significant differences (Dunnet's *post-hoc* test). ^*^P < 0.05; ^**^P < 0.01.

Small fragments of tobacco calluses were weighed and exposed to increasing concentrations of AM synthetic peptide. After growing in the dark for 30 days (Figure 1A), calluses were weighed again and their growth rate for that period of time was calculated. One-way ANOVA indicated that the treatment was significantly efficacious (*p* < 0.05). The higher dose of AM (10 nM) induced a 36.46% weight increase (*p* < 0.01, Dunnet *post-hoc* test) in comparison with the untreated control group (Figure 1B). The AM precursor, Gly-AM, had no effect on callus growth at any concentration (Figure 1B shows only the highest concentration tested). To investigate whether this AM-induced weight increase was due to water retention or to a real biomass increase, calluses were dried and the statistically significant weight difference (22.71%) was maintained (Figure 1C), thus demonstrating that AM elicits a real growth of the tobacco calluses.

### Transgenic tobacco plants expressing AM grow larger than controls

**Figure 2:**
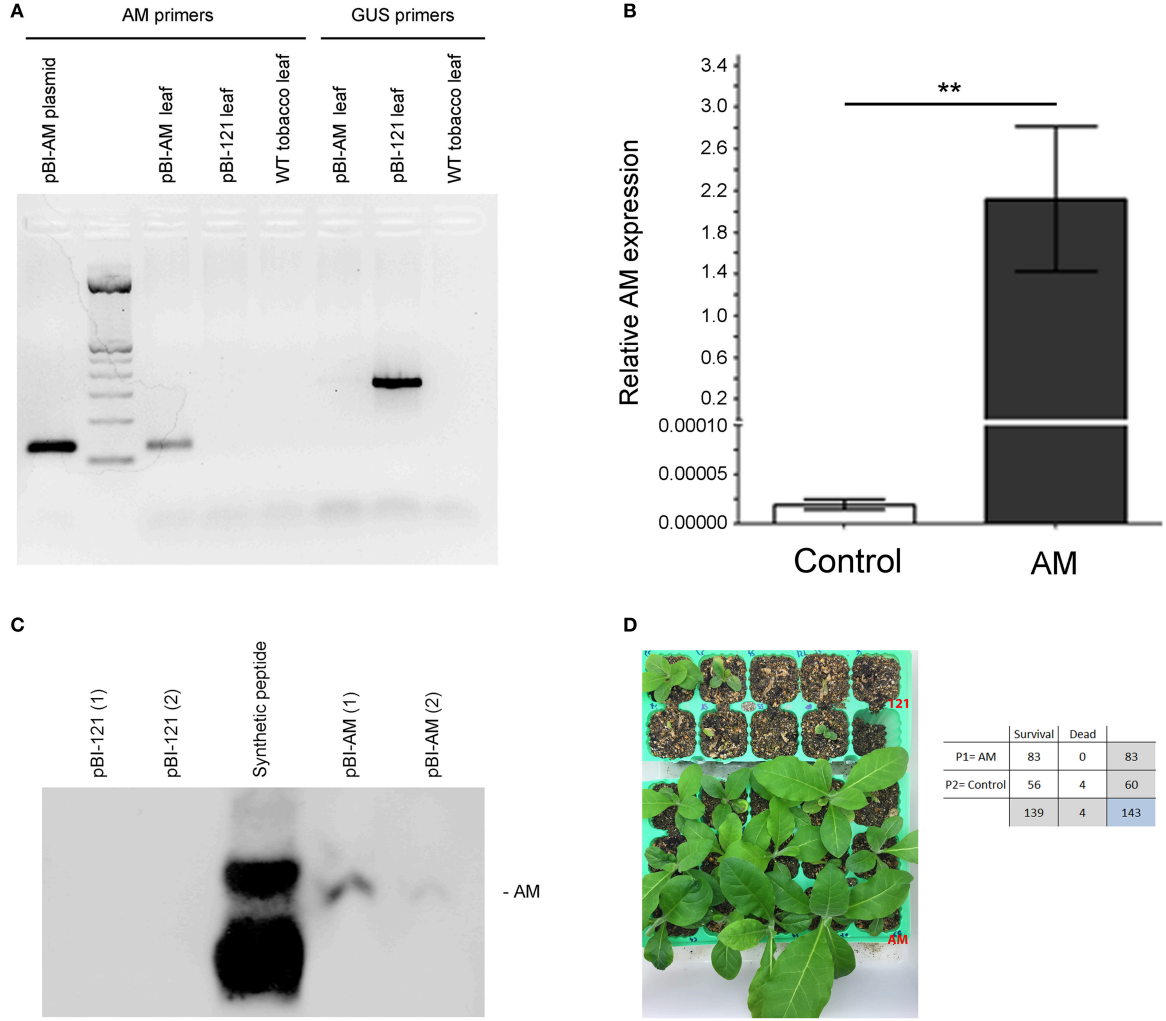
**Confirmation of transgene acquisition and function**. DNA was extracted from leaves of young transgenic tobacco plants and subjected to PCR with specific primers for either AM or GUS **(A)**. AM mRNA expression was measured through qRT-PCR in the same leaf extracts **(B)**. Bars represent the mean ± *SD* of all specimens (*n* = 5 per genotype). Asterisks indicate statistically significant differences (Mann-Whitney's *U*-test). ^**^P < 0.01. Protein expression was also measured on leaf extracts through Western blotting **(C)**. AM synthetic peptide (12 ng) was loaded as a positive control. Transgenic plants for AM (AM, lower area) and those transformed with plasmid pBI-121 as a control (121, upper area) were transferred from *in vitro* conditions to real soil **(D)**. Several pBI-121 plants are shown in the photograph dead or dying whereas all the pBI-AM plants are healthy and growing fast. The table shows the frequencies of dead and surviving plants for both genotypes. χ^2^-test, *p* = 0.0014.

**Figure 3:**
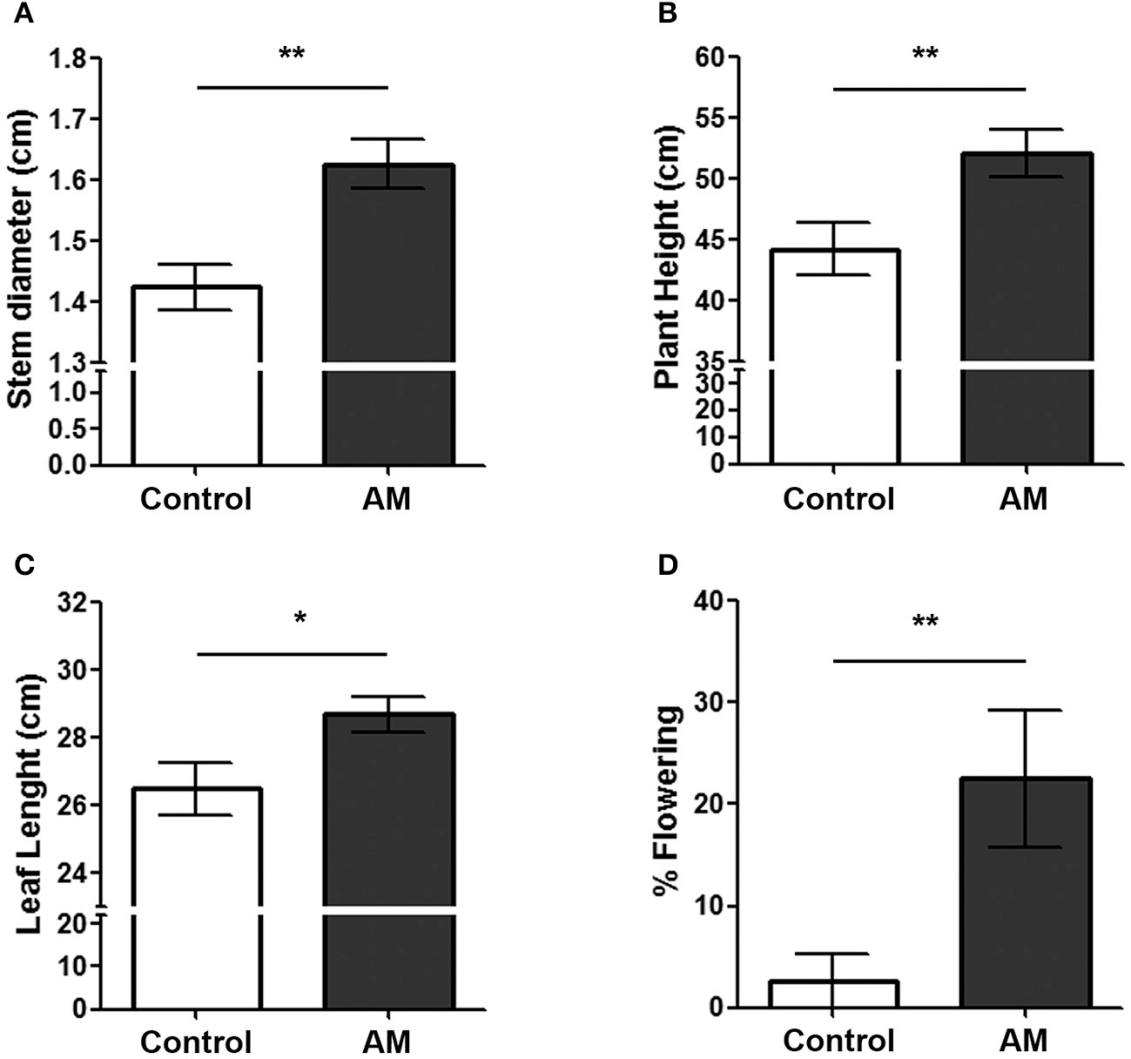
**Anatomical measurements of AM transgenic plants (AM) and those transformed with plasmid pBI-121 as a control**. Stem diameter **(A)**, plant height **(B)**, leaf length **(C)**, and the percentage of flowering plants **(D)** were measured 5 weeks after transplantation in regular soil. Bars represent the mean ± *SD* of all specimens (*n* = 36 for controls; *n* = 38 for AM). Asterisks represent statistically significant differences between genotypes (Student's *t*-test). ^*^p < 0.05; ^**^p < 0.01.

Transgenic plants were generated using plasmid pBI-AM, or pBI-121 as a control plasmid. All plants were genotyped by PCR with specific primers for AM and β-glucuronidase, GUS (Figure 2A). To confirm that AM was expressed in the transgenic plants, qRT-PCR (Figure 2B), and Western blotting (Figure 2C) were performed on leaf extracts showing a high expression of both the mRNA and the peptide in transformed plants. For all experiments, only primary transformants were used, representing independent plasmid insertion events. A first physiological observation was the improved survival of AM transgenic plants in the critical step of the transfer from agarose-based medium to real soil. Survival for AM transgenic plants was 13.25% higher than for pBI-121 control plants (Figure 2D). Furthermore, several morphological parameters were measured at different times during the plant's life cycle. Stem diameter, plant height, and leaf length were significantly larger (14.18, 17.72, and 8.28%, respectively) in AM transgenic plants than in their pBI-121 transgenic counterparts at all times (Figures 3A–C) whereas there was no difference in internodal length or in the total number of leaves per plant (results not shown). Another parameter where statistically significant differences were found was in the flowering time, which occurred earlier in AM transgenic plants. For instance, at 5 weeks after transplantation, many more (755.00%) AM plants had flowered than controls (Figure 3D).

**Figure 4:**
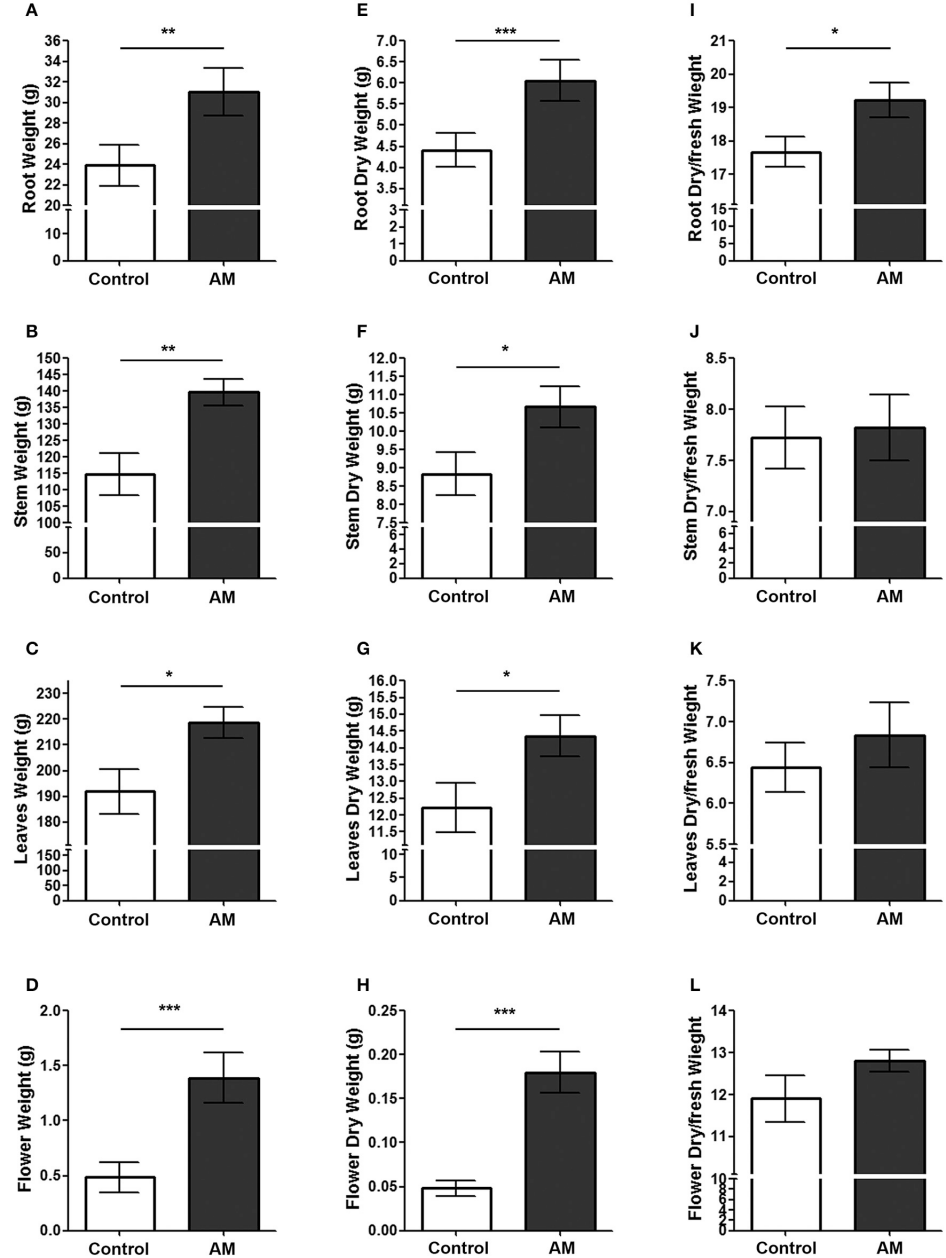
**Weight parameters after flowering**. The fresh **(A–D)** and dry **(E–H)** weight, as well as the dry/fresh ratio **(I–L)** was determined for the root system **(A,E,I)**, the stems **(B,F,J)**, the leaves **(C,G,K)**, and the flowers **(D,H,L)** of AM transgenic plants (AM) and those transformed with plasmid pBI-121 as a control. Bars represent the mean ± *SD* of all specimens (*n* = 36 for controls; *n* = 38 for AM). Asterisks represent statistically significant differences (Student's *t*-test). ^*^p < 0.05; ^**^p < 0.01; ^***^p < 0.001.

After the first floral buds opened but before complete flower maturation, the plants' total weight and the weight of particular organs were measured. The weight of AM-expressing transgenic plants and their particular organs: roots (29.89%), stems (21.66%), leaves (14.01%), and flowers (187.09%), was significantly heavier than this of the pBI-121 controls (Figures 4A–D). These differences were maintained (roots 37.18; stems 20.68; leaves 17.46; flowers 271.13%) after drying the different organs (Figures 4E–H), demonstrating a true increase in plant biomass. Interestingly, when the dry/fresh weight ratio was calculated it was the same between genotypes for the stems, leaves, and flowers (Figures 4J–L) but in the case of the roots, the ratio was significantly higher (8.80%) in the AM transgenic plants (Figure 4I), indicating that AM aids in the development of a larger and denser root system. The mean general increase in dry biomass for the whole plant was 21.31% when compared with pBI-121 controls.

### Transgenic tobacco plants expressing AM possess a larger root system but maintain a normal histological structure

**Figure 5:**
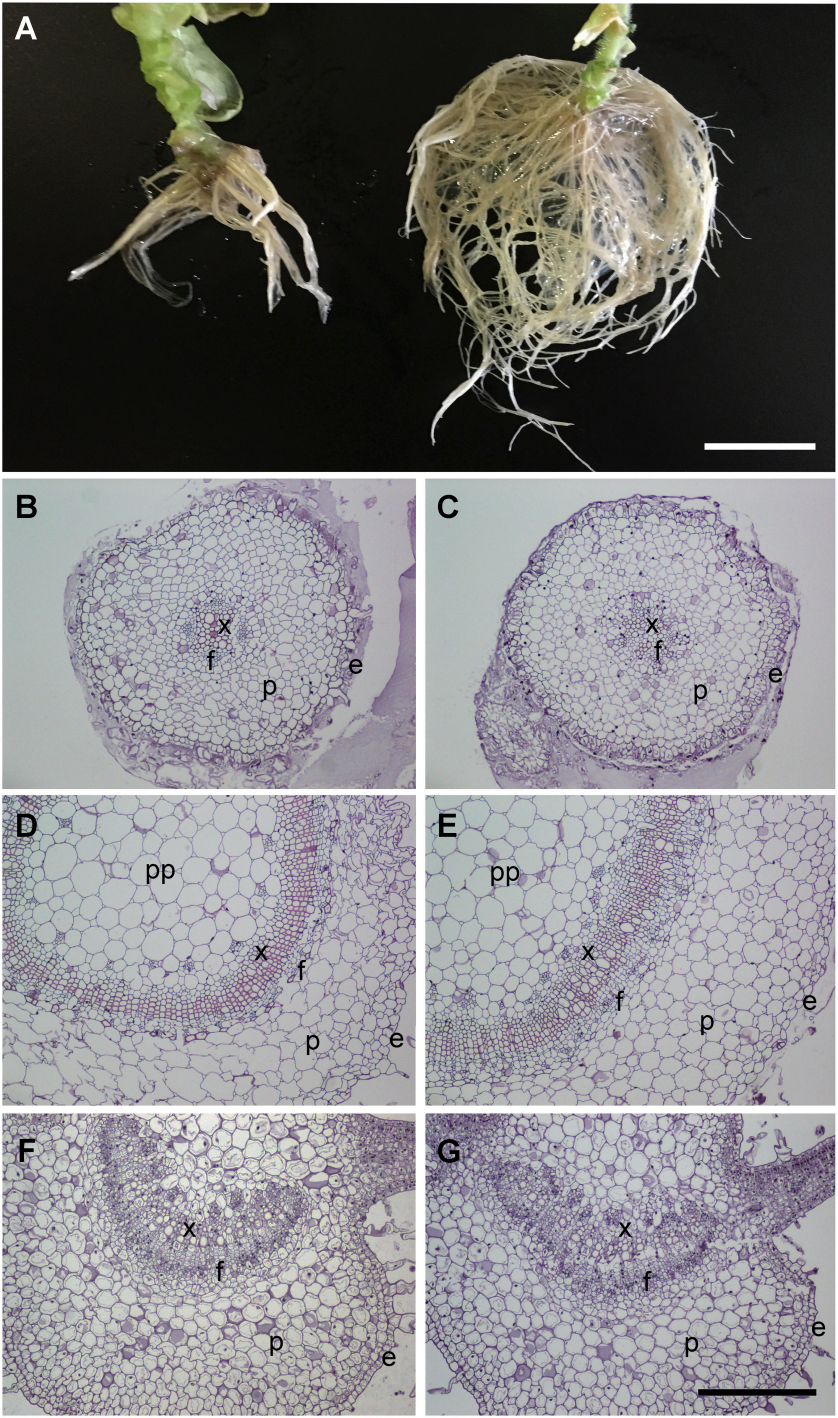
**Morphology of transgenic plants**. Macroscopic view of the root systems **(A)** for a representative AM transgenic plant (right) and a pBI-121 control plant (left). Histological appearance of roots **(B,C)**, stems **(D,E)**, and leaves **(F,G)** of AM transgenic plants **(C,E,G)** and those transformed with plasmid pBI-121 as a control **(B,D,F)**. e, epidermis; f, phloem; p, parenchyma; pp, pith parenchyma; x, xylem. Bar in **(A)** = 5.0 mm. Bar for **(B–G)** = 400 μm.

Given the differences in organ weight, we investigated the morphology of these organs in more detail. The aspect of the leaves and stems, although larger (as described above), were morphologically similar between AM transgenic plants and normal controls, but the root system was strikingly different containing more numerous and thinner roots than the controls (Figure 5A). To check whether there were changes in the histological structure of these plants, sections of roots (Figures 5B,C), stems (Figures 5D,E), and leaves (Figures 5F,G) were studied. Apart from differences in diameter, the general structure of these organs was similar between genotypes.

### Transgenic tobacco plants expressing AM do not lose basic biochemical components

**Figure 6:**
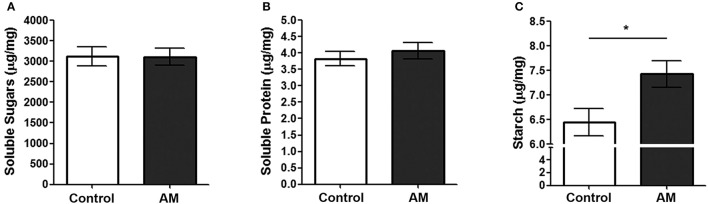
**Biochemical markers in the leaves of both genotypes**. Soluble sugars **(A)**, soluble proteins **(B)**, and starch contents **(C)** were measured in the leaves of AM transgenic plants (AM) and in pBI-121 controls. Bars represent the mean ± *SD* of all specimens (*n* = 35 for controls; *n* = 38 for AM). Asterisks represent statistically significant differences (Student's *t*-test). ^*^p < 0.05.

To test whether the growth increase observed in AM-expressing plants resulted in a detrimental decrease of biochemical components, we measured the levels of soluble sugars, proteins, and starch in the leaves of both genotypes at the end of the plant's life cycle. No differences were observed for soluble sugars and proteins (Figures 6A,B) but there was a 15.22% significant increase in starch in the leaves of AM transgenic tobacco plants when compared to their controls (Figure 6C).

### Overexpression of AM co-opts key intracellular cell cycle controllers

**Figure 7:**
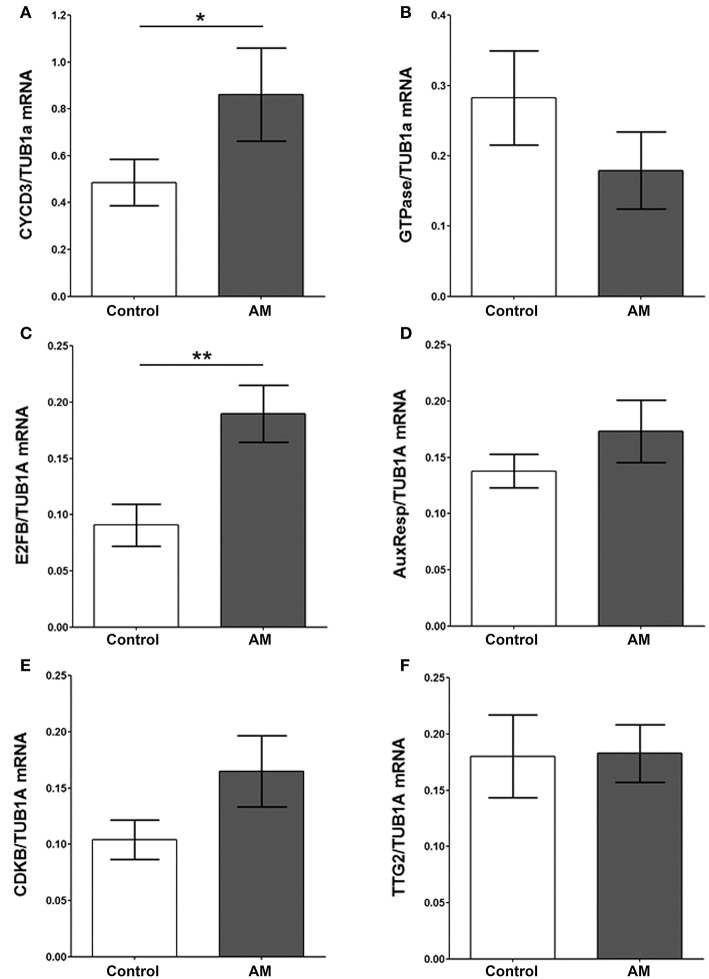
**Gene expression of different transcription factors**. The expression level of several tobacco transcription factors, CycD3 **(A)**, GTPase **(B)**, E2FB **(C)**, AuxResp **(D)**, CDKB **(E)**, and TTG2 **(F)**, was determined by qRT-PCR in leaf extracts of pBI-121 controls and in AM transgenic plants (AM). All values were relativized by the levels of the house keeping gene Tubulin α1. Bars represent the mean ± *SD* of all specimens (*n* = 10 per genotype). Asterisks represent statistically significant differences (Mann-Whitney's *U*-test). ^*^p < 0.05; ^**^p < 0.01.

To better understand AM's mechanism of action on plant cells, we investigated the gene expression of several key cyclins and transcription factors related to cell cycle modulation on plants of both genotypes. There was a statistically significant 81.29% increase of cyclin D3 (Cyc D3) (Figure 7A) and a 109.04% increase of transcription factor E2FB (Figure 7C) expression in the transgenic plants when compared with the pBI-121 controls. Other tobacco intracellular signals, such as GTPase (Figure 7B), auxin responsive-like gene (AuxResp) (Figure 7D), cyclin-dependent kinase B1 (CDK B1) (Figure 7E), and transcription factor Transparent Testa Glabra 2 (TTG2) (Figure 7F) were not significantly different between genotypes.

### A search of the tobacco genome did not identify an AM ortholog

We performed a search of tobacco's predicted proteome looking for peptides with some sequence homology to any of the available vertebrate AM sequences and none was found using the default value threshold parameters of the software packages. Since the more relevant features of AM are the intramolecular 6-amino acid ring: Cys-X-X-X-X-Cys, and the terminal amide group represented by the sequence Tyr-Gly-Arg-Arg-Arg-Arg-Arg (Lopez and Martinez, 2002), we also performed a search for molecules containing these motifs. Although some proteins contained these sequences, none of their predicted post-translational processing or intracellular distribution was compatible with a secreted peptide hormone similar to mammalian AM.

## Discussion

In this study we have shown that mammalian AM increases tobacco's biomass both in tissue culture and in transgenic plants. These observations are in line with previous publications where another animal peptide, epidermal growth factor, was shown to induce the growth of corn mesocotyls *in vitro* (Kato et al., 1995). The search of the tobacco's genome for an ortholog of AM in plants did not produce any obvious candidate, thus we have to hypothesize that the action of mammalian AM on plant's tissues must be mediated by the co-option of an unknown receptor system involved in the plant's growth. Future studies should focus in identifying this receptor system and its intrinsic agonists.

In this study we have analyzed primary transformant plants. Transgenesis is a technique that relies on the random insertion of the vector into the plant's genome. Therefore, a phenotype could be the result of altering the expression or epigenetic status of neighboring genes rather than of the expression of the inserted gene (Kohli et al., 2006). In that regard it is very important to study as many founders as possible to ensure that the common phenotype is independent of the insertion locus. This is why we believe that, at this stage, it is better to study primary transformants rather than downstream generations. Future studies should analyze transgenic lines to demonstrate that the transgene is stable and that the gene expression can be transferred from generation to generation.

Our experiments on tobacco calluses demonstrated that AM acts as a proliferation factor in this setting. In mammals, AM growth-related actions are mediated by intracellular elevations of cAMP and activation of mitogen-activated protein kinase cascades, among others (Larrayoz et al., 2014). These pathways are also present in vascular plants and both of them regulate plant cell proliferation (Xu and Zhang, 2015; Sabetta et al., 2016). Cell cycle, both in animals and plants, is driven by several regulatory proteins designated as cyclins and their cyclin-dependent kinases (CDK), whose expression often depends on plant hormones, growth conditions, and developmental stages (Espinosa-Ruiz et al., 2004). Cell cycle-dependent phosphorylation of RB-related protein by CDKs results in the release of active E2F transcription factors that induce a wave of transcriptional activity in the cell (Nakagami et al., 2002). In our transcription factor expression study we have found that AM transgenic plants had a higher expression of the CycD3 and the transcription factor E2FB. Cyclins of the D group control the onset of cell division and responses to extracellular signals during G1 phase and CycD3 is expressed only in organs and tissues where cell division is taking place (Qu and Wang, 2007). E2FB stimulates cell division by promoting both G1-to-S and G2-to-M transitions, leading to shorter duplication times. E2FB abundance and stability is increased by exogenously applied auxin (Magyar et al., 2005). Our present results show that these signaling molecules are also activated by AM. On the other hand, other genes which are also related to the auxin pathway, such as TTG2 (Zhu et al., 2013), auxin response-like gene (Gonzalez-Perez et al., 2014), or the GTPase did not respond to AM overexpression. This panorama suggests a specific intracellular pathway for AM signaling that shares some components but not others with classic growth promoters such as auxin. More studies are needed to draw the complete circuitry of AM's intracellular mediators.

Nevertheless, a factor that induces mitosis in plants may result in disorganized growth and the generation of tumor-like tissue. This is the well-known behavior of the Ti plasmid of *A. tumefaciens* which results in the uncontrolled growth of plant cells and the apparition of crown gall disease in infected plants (Gohlke and Deeken, 2014). This is why it was important to generate AM transgenic plants and investigate whether the overexpression of this peptide influences growth in the whole plant and whether it has an undesirable impact on plant morphology and development. In our case, the mammalian peptide generated larger and heavier plants but preserving plant architecture and biochemical composition, suggesting that this molecule works as a *bona fide* growth factor for plants, not influencing pattern formation. This is important if we want to apply this factor to improve commercially relevant crops.

Another important feature was the improved survival of plantlets in the critically stressful step of transplantation from *in vitro* culture to real soil condition. Many growth factors act as survival and stress-reducing factors, as well, due to their activity modulating apoptosis and other intracellular preservation pathways (Collins et al., 1994). In mammals, AM has been shown to exert a survival function at the level of individual cells (Cuttitta et al., 2002) and also at the organism level (Caron and Smithies, 2001), and to be involved in stress reduction (Fernandez et al., 2008). Future studies would address whether AM transgenic plants are more resistant to typical plant stressors such as drought or infectious pathologies. In this regard, we should remember that AM has antimicrobial properties due to its alpha-helical structure and an ability to insert itself in the pathogen's membrane, opening up hydrophilic pores that result in bacterial demise (Allaker et al., 2006; Martinez et al., 2006). This feature could be an added bonus for AM transgenic crops.

An interesting observation was that the roots of AM transgenic plants were more numerous and had more dry matter proportion than their control counterparts whereas all other organs had similar percentages. This suggests that AM has a special impact on the root, making it not only to grow larger but also increasing the amount of solid biological tissue. Other plant growth factors and intracellular signals have preferential activity on specific organs. For instance, the HHO2 (hypersensitivity to low phosphate-elicited primary root shortening 1 homolog 2) transcription factor regulates root growth without influencing other plant organs (Nagarajan et al., 2016). Our observation that AM may share part of the auxin transcription factor machinery suggests a special regulation of the root system. Perhaps AM could be used to facilitate the development of a robust root system for plants, which is essential for successful cultivation and crop improvement.

Although AM-transgenic crops are not intended for human or animal consumption, if some of these plants find their way accidentally into the food chain, the presence of AM should be harmless. AM is naturally present at high concentration in human and mammalian milk, where it contributes to the healthy development of the infant's gut (Pio et al., 2000). In addition, dietary AM is rapidly degraded by pancreatic exo- and endo-peptidases into individual amino acids, thus destroying its signaling properties.

Preliminary experiments ongoing in our laboratory show similar results in the growth of other plant species, including vascular plants as well as unicellular microalgae, suggesting that AM could be used as a general growth promoter for increasing plant biomass through biotechnological manipulation of different crops.

In conclusion, we have shown that mammalian AM improves tobacco plant survival and growth without modifying its general architecture and biochemical composition. These observations suggest broad applications for improving crop productivity through biotechnology.

## Author contributions

RP and MN performed experiments and analyzed data; AM designed and supervised the experiments and wrote the manuscript; all authors read and approved the final version of the manuscript.

## Funding

This study was funded in part by a grant from Agencia de Desarrollo Económico de La Rioja (ADER), exp n° 2014-I-DPT-00047.

### Conflict of interest statement

RP and MN are former employees of Biomass Booster, Inc. AM is inventor of several patents regarding the beneficial effects of adrenomedullin on crop production and owns stock on Biomass Booster, Inc.
